# Nitrogen balance along a northern boreal forest fire chronosequence

**DOI:** 10.1371/journal.pone.0174720

**Published:** 2017-03-30

**Authors:** Marjo Palviainen, Jukka Pumpanen, Frank Berninger, Kaisa Ritala, Baoli Duan, Jussi Heinonsalo, Hui Sun, Egle Köster, Kajar Köster

**Affiliations:** 1 Department of Forest Sciences, University of Helsinki, Helsinki, Finland; 2 Department of Environmental and Biological Sciences, University of Eastern Finland, Kuopio, Finland; 3 Institute of Mountain Hazards and Environment, Chengdu, China; 4 Department of Food and Environmental Sciences, University of Helsinki, Helsinki, Finland; 5 Collaborative Innovation Center of Sustainable Forestry in Southern China, College of Forestry, Nanjing Forestry University, Nanjing, China; Pacific Northwest National Laboratory, UNITED STATES

## Abstract

Fire is a major natural disturbance factor in boreal forests, and the frequency of forest fires is predicted to increase due to climate change. Nitrogen (N) is a key determinant of carbon sequestration in boreal forests because the shortage of N limits tree growth. We studied changes in N pools and fluxes, and the overall N balance across a 155-year non stand-replacing fire chronosequence in sub-arctic *Pinus sylvestris* forests in Finland. Two years after the fire, total ecosystem N pool was 622 kg ha^-1^ of which 16% was in the vegetation, 8% in the dead biomass and 76% in the soil. 155 years after the fire, total N pool was 960 kg ha^-1^, with 27% in the vegetation, 3% in the dead biomass and 69% in the soil. This implies an annual accumulation rate of 2.28 kg ha^-1^ which was distributed equally between soil and biomass. The observed changes in N pools were consistent with the computed N balance +2.11 kg ha^-1^ yr^-1^ over the 155-year post-fire period. Nitrogen deposition was an important component of the N balance. The biological N fixation increased with succession and constituted 9% of the total N input during the 155 post-fire years. N_2_O fluxes were negligible (≤ 0.01 kg ha^-1^ yr^-1^) and did not differ among post-fire age classes. The number and intensity of microbial genes involved in N cycling were lower at the site 60 years after fire compared to the youngest and the oldest sites indicating potential differences in soil N cycling processes. The results suggest that in sub-arctic pine forests, the non-stand-replacing, intermediate-severity fires decrease considerably N pools in biomass but changes in soil and total ecosystem N pools are slight. Current fire-return interval does not seem to pose a great threat to ecosystem productivity and N status in these sub-arctic forests.

## Introduction

Fire is a major natural disturbance factor in boreal forests, with approximately 1% of boreal forests burned annually [1]. The frequency of forest fires is predicted to increase due to climate change in the boreal zone [2, 3]. As the boreal forests are the second largest biome on Earth, encompassing ~30% of the global forest area [1], increases in the annual area burned could have significant implications for carbon (C) and nitrogen (N) cycles [2, 4].

The availability of N plays a central role in regulating biomass production, C allocation, and organic matter decomposition in boreal forests [5]. Consequently, N is a key determinant of C pools and sequestration, especially in N-limited boreal forests [6, 7]. Fires cause N loss from ecosystems through oxidation and volatilization of N stored in biomass, woody debris and litter [8, 9], and through post-fire N losses via leaching [10] and nitrous oxide (N_2_O) emissions [11, 12, 13]. Part of the N stored in organic layer is also lost, but the N pool of mineral soil usually remains quite unchanged [9, 14, 15, 16, 17]. The increase in N leaching generally disappears within a few years after fire [18], and leaching losses are small compared to the N volatilization during a fire as well as compared to soil N pools [19]. The N_2_O fluxes are low in boreal upland forests [20], but they may change during post-fire succession [11, 12, 13] due to changes in soil temperature, moisture and pH [21]. Soil temperature increases after forest fire as the dark surface of a burned site effectively absorbs solar radiation [22, 23]. The fire also affects soil moisture conditions because charred humus has smaller water holding capacity and ability to hinder evaporation than unburned humus [22, 23]. Post-fire increases in soil temperature and concentrations of NH_4_ and NO_3_ [24, 25] may increase N_2_O emissions whereas decreased soil moisture may have an opposite effect [26, 27].

To sustain forest productivity and the ability to sequester C, the N losses after disturbance must be balanced by N inputs through atmospheric deposition and biological N fixation. N balance in boreal forests may be poorly buffered against forest fires because atmospheric N deposition (at maximum 10 kg ha^-1^ yr^-1^) and biological N-fixation are small (0.3–4 kg ha^-1^ yr^-1^) in these ecosystems [28, 29, 30]. Feathermoss species that support epiphytic cyanobacteria represent the primary source of biological N fixation in the late succession boreal coniferous forests [29]. Also, N-fixation by free-living and associative bacteria occurs in forest soils [28, 31]. Despite the potential importance of N-fixing bacteria, studies on their ecology and N-fixing activity are scarce [32], and the influence of disturbances and successional stage on N fixation in boreal forests is poorly known. Fires affect the activity of biological N-fixation by partly or completely destroying the moss layer vegetation where most of the N-fixing cyanobacteria live [33]. Increased soil pH and temperature after fire may enhance N fixation [31, 34] whereas increased availability of inorganic N may have a suppressing effect [33].

The recently developed GeoChip microarray, a high through-put approach to study the potential environmental microbial community functions, offers the possibility to assess microbial genes involving in different biogeochemical processes, such as C and N cycles [35]. Quantification the presence of microbial genes involved in the cycling of N in forest soils has been used to assess microbial functional group abundance, metabolic activity and changes in N processes [36]. The impact of forest fires on microbial biomass and community structure have been studied to a some extent [23], but very little is known how genes involved in N cycling are influenced by forest fires or affect the rates of N processes [37]. The link between processes regulating the N cycling at the gene level and N pool sizes and fluxes is important to form but is generally challenging to establish due to scale differences, natural variation in the field and high analysis costs of molecular biological tools like microarrays and metagenomics.

Forest fires affect N pools and fluxes by changing vegetation, the quantity and quality of soil organic matter, abiotic factors and the biomass, activity and community structure of soil microbes [37, 38, 39]. Responses have been variable, as they are dependent on fire intensity, initial soil N pools, atmospheric deposition of N, climatic conditions and time since the fire [23, 24, 25, 40]. The non-stand-replacing, intermediate-severity fires are the dominant fire type in Eurasian boreal forests [41]. Although forest fires have a large effect on N dynamics, there are few quantitative data available on N pools and fluxes through post-fire succession in sub-arctic boreal forests. Review studies indicate that most of the research has focused on short-term responses, and little is known about the effects of forest fire on N cycling through succession [24, 25].

The aim of this study was to examine changes in the pools, fluxes and balance of N along a 155-year fire chronosequence in sub-arctic Scots pine (*Pinus sylvestris* L.) stands, and to investigate how the number of microbial genes associated with N cycling are linked to N dynamics. We hypothesize that 1) forest fires considerably decrease ecosystem N pools and change the distribution of N among different components, 2) it takes a long time before atmospheric deposition and N fixation inputs replace N losses, 3) the importance of deposition increases after forest fire since N-fixing moss layer is destroyed, 4) even small N-fixation rates have large impact on the N balance in these N-limited subarctic ecosystems where the atmospheric N deposition is among the lowest in Europe [42] and 5) changes in N fluxes caused by fires are reflected by the changes in the number and intensity of microbial genes related to N cycling.

## Materials and methods

### Study area

The study area is located in Värriö Strict Nature Reserve in Finland (67°46′ N, 29°35′ E), in northern boreal subarctic coniferous forests. The area is situated 250 km north of the Arctic Circle in Eastern Lapland, near to the northern timberline at an average of 300 m altitude. The University of Helsinki has a Field Station in Värriö and the University of Helsinki has a permanent contract for using the land area owned by the Finnish Forest and Park Service for experimental use and teaching purposes. This field study did not involve endangered or protected species. The main tree species is Scots pine (*Pinus sylvestris*) (Table 1). Field layer vegetation (vascular plants) is dominated by *Vaccinium myrtillus* L., *Vaccinium vitis-idaea* L., *Empetrum nigrum* L. and *Calluna vulgaris* L., and the bottom layer (mosses and lichens) by *Hylocomium splendens* (Hedw.) Schimp., *Dicranum* Hedw. sp. and *Cladina* (Nyl.) Nyl. sp. (Table 2). The soil is sandy till and classified as haplic podzol [43], (Table 1). The particle size of mineral soil in the area is coarse or fine sand, with the proportion of silt <10% [39]. The thickness of F-horizon varies from 0 to 2.5 cm and the thickness of O-horizon from 0 to 1.2 cm. The thicknesses of A- and B-horizons range from 1.0 to 4.5 cm and from 1.0 to 6.0 cm, respectively [39]. The climate is sub-continental, with long-term (1971–2000) mean annual air temperature of −0.9°C and mean annual precipitation of 592 mm [44]. The soil is covered by snow for the majority of the year (on average 209 days). The average length of growing season is 114 days; during that period mean precipitation is 250 mm and mean temperature approximately 1.2°C. The soil has no underlying permafrost.

**Table 1:** Soil and tree characteristics of study areas.

Years after fire	Year of last fire	Location	Soil	pH organic layer /mineral soil	Tree species composition Pine/Birch (%)	No of trees / biomass of trees survived from fire (%)	No of trees ha^-1^	Mean tree diameter(1.3 m)	Mean tree height (m)
2	2009	67˚47.62′ N, 29˚40.97′ E	Sandy till	4.0/4.4	92/8	42/60	1438	9	11.9
42	1969	67˚46.99′ N, 29˚41.96′ E	Sandy till	3.6/4.4	100/0	20/45	1025	9	8.8
60	1951	67˚45.76′ N, 29˚37.06′ E	Sandy till	3.8/4.3	95/5	n.d.	788	11	9.5
152	1859	67˚45.12′ N, 29˚37.87′ E	Sandy till	3.7/4.4	96/4	n.d.	213	30	17.0

n.d. = not determined

**Table 2:** The mean (SE) coverage of mosses and lichens in the study areas. The coverage was assessed from 20 (1 m×1m) quadrats from each plot.

Plot	*Dicranum* sp.	*Pleurozium schreberi*	*Polytrichum*sp.	Other mosses	*Cladina rangiferina*	*Cladina arbuscula*	*Cladonia stellaris*	Other lichens
5			3.6 (0.7)					
45		11.1 (4.0)	0.2 (0.1)		6.4 (5.0)	2.7 (1.6)		0.1 (0.1)
63	14.3 (5.3)	56.4 (8.4)		3.1 (2.1)	2.8 (1.5)	2.9 (1.7)	0.9 (0.5)	
155	60.9 (6.7)	24.8 (7.1)	0.5 (0.5)		6.6 (1.6)	7.7 (1.7)	0.1 (0.1)	

In 2011, the sample plots were established in areas where a natural forest fire occurred 2, 42, 60 and 152 years ago (Table 1). The living and dead biomass were re-measured from the same sample plots three years later when 5, 45, 63 and 155 years had elapsed from the fire. There were three sample plots in the youngest age class and four sample plots in all the other age classes. All sample plots were circular with an area of 400 m^2^ and they were situated 200–500 m apart from each other. The size of the total study area where the sample plots were established was 18 km^2^. Dendrochronological dating was used for determining the time since the last fire [39]. Fires were non-stand replacing in all study areas. Based on visual estimations, 42% and 20% of the trees survived from the fire in 2009 and 1969 burned areas, respectively. The exact number of surviving trees or the exact time of dead of trees is not known in the two oldest age classes because the determination of the initial pre-fire stand conditions was not possible because dead trees were heavily decayed and they might even be not anymore detectable. Also, dendrochronological dating was not possible on the decayed wood of many fallen trees in the areas. Forest fires occur in Fennoscandian boreal forests with intervals of 50–150 years [45, 46]. Consequently, as in many boreal fire chronosequence studies, we were not able to find an unburnt control, since all the landscape has burned at some point in time [47]. Therefore, the 155-year-old area was used as a reference area. A detailed description of the study areas are presented in previous publications [13, 37, 39].

### N pools in the living above-ground tree biomass

Tree height, breast height diameter (at 1.3 m height), crown height and crown diameter of every tree were measured in each plot. The biomass of stem wood, stem bark, living branches, dead branches and needles was estimated by using allometric biomass functions [48]. The N pools in tree biomass were calculated by multiplying the dry weights of the different tree compartments with the corresponding N concentrations (Table 3). The difference between the N content of the tree stands two years after the forest fire and 155 years after the forest fire was regarded as an accumulation of N.

**Table 3:** Nitrogen concentrations of different biomass compartments used for biomass N pool calculations.

	N (% of dry weight)	References
Stemwood	0.09	[49, 50, 51, 52]
Stembark	0.32	[49, 50, 51, 52]
Living branches	0.51	[49, 51, 52]
Dead branches	0.26	[49, 51, 52]
Needles (5-year plots)	1.15	Measured on site
Needles (45-year plots)	1.08	Measured on site
Needles (65-year plots)	1.17	Measured on site
Needles (155-year plots)	1.01	Measured on site
Roots > 10 mm	0.29	[53]
Fine roots < 2 mm	0.40	[53]
Standing dead trees		
Decay class 1	0.17	[54]
Decay class 2	0.21	[54]
Decay class 3	0.20	[54]
Decay class 4	0.23	[54]
Decay class 5	0.23	[54]
Lying dead trees		
Decay class 1	0.17	[54]
Decay class 2	0.24	[54]
Decay class 3	0.33	[54]
Decay class 4	0.45	[54]
Decay class 5	0.49	[54]
Ground vegetation	0.95	Measured on site

### N pools in dead tree biomass

The diameter, length and degree of decay of dead trees were determined on all plots, and their biomass was calculated by using the biomass functions of [48]. Five decay classes were distinguished based on the structural integrity, wood texture and the presence of bark [55]. The following decay classes were used: I) recently dead, wood still hard, knife blade penetrates a few millimeters, bark normally intact, II) weakly decayed, wood of outer layers of stem has started to soften, wood still fairly hard, knife blade penetrates < 2 cm, loose bark, III) medium decayed, wood of outer layers of stem fairly soft, core still hard, knife blade penetrates 2–5 cm, usually without bark, IV) very decayed, wood soft throughout the log, no hard core, knife blade penetrates all the way and V) almost decomposed, wood very soft, fragmented, breaks up easily by hand, often overgrown with lichens, mosses and dwarf shrubs. The N pools in dead tree biomass were calculated by multiplying the dry weights of the different decay classes with the corresponding N concentrations (Table 3).

### N pools in ground vegetation

Above-ground biomass of ground vegetation was harvested from 5 squares (20 cm × 20 cm each) located systematically inside the sample plots. In the laboratory samples were divided into bottom layer vegetation (mosses and lichens) and field layer vegetation (dwarf shrubs, grasses and herbs), after which biomass fractions were dried (60°C, 48 h), weighed, ground and the N concentrations were determined with a Vario Max CN-analyzer (Elementar Analysensysteme GmbH, Germany).

### N pools in the below-ground biomass of trees and ground vegetation

The living coarse root (diameter >10 mm) biomass was calculated by using biomass functions [48]. The biomass of fine (diameter < 2 mm) and small (2–10 mm) roots was determined by the core method. From each plot, 10 cylindrical cores (diameter 50 mm) were taken from organic layer and upper 15 cm mineral soil layer. The roots were extracted from cores by hand and divided into living and dead roots based on elasticity and toughness [56]. Root samples were dried (60°C, 48 h), weighed and the N contents were calculated by multiplying dry masses with N concentrations (Table 3).

### Soil N pools

From each sample plot, 10 cylindrical cores (diameter 50 mm) were taken from upper 15 cm soil layer [39]. Organic layer samples were divided into fermentation (F) and humified (H) layers, and the mineral soil samples into eluvial (E-horizon) and illuvial (B-horizon) layers. The samples were dried (60°C, 24 h), sieved through a 2 mm sieve, and ground before the analysis. Subsamples were taken for dry mass determination at 105°C. Nitrogen concentrations were analyzed from the homogenized samples with an elemental analyzer (Vario Max CN elemental analyser, Elementar Analysensysteme GmbH, Germany). The N pool, *N*_*p*_ (kg ha^-1^) of each soil horizon was calculated using the following formula:

Np=Nconc×BD×L×100,

where *N*_*conc*_ is the N concentration (mg g^-1^ soil), BD is the measured bulk density of the less than 2 mm fraction (g cm^-3^) and *L* is the thickness of the soil layer (cm). The total soil N pool was calculated as the sum of N pools of all horizons.

### Atmospheric N deposition

The total annual N deposition to the study area was calculated as the sum of wet and dry deposition. The wet deposition data for the years 1970–2011 were compiled from the databases of Finnish Meteorological Institute (FMI) (measurements in Värriö during the years 1990–1995 and 1999–2003), The European Monitoring and Evaluation Programme (EMEP) (measurements in Oulanka and Pallas stations during the years 1990–2013) and Finnish Environmental Institute (measurements in Sodankylä station during the years 1970–1982). FMI and EMEP databases did not contain organic N deposition, and for those years, the organic N deposition was assumed to account for 28% of total N deposition which is the long-term mean value in Finland [57]. The values for dry deposition were taken from Flechard et al. [58]. There are no measured N deposition data in Lapland before the year 1970. Historical N deposition was estimated by using the values that are presented by Ruoho-Airola et al. [59]. Nitrogen deposition in 1960 was estimated to be 65% of the 1970 level, N deposition in 1950 was 50% of the 1970 level, N deposition in 1925 was 38% of the 1970 level, N deposition in 1900 was 30% of the 1970 level, and N deposition in 1850 was 25% of the 1970 level [59]. The depositions between the reference years were linearly interpolated.

### N fixation

Biological N-fixation was investigated in the study areas (Table 1) by collecting 10 cylinder samples (diameter 0.05 m, height 0.12 m) containing ground vegetation and organic layer four times during the summer of 2014 [60]. Nitrogen fixation was determined by using acetylene reduction method [33, 61] which measures the reduction of acetylene to ethylene by the nitrogenase enzyme. The samples were inserted into glass vials in the sampling day, injected with acetylene and incubated for 24 hours in field conditions in the study plots. During the experiment in June, July, at the beginning of August and at the end of August, the air temperatures were 17°C, 23°C, 24°C and 10°C, respectively. Ethylene concentrations were measured by a gas chromatograph equipped with a flame ionization detector (450-GC, Varian, California, USA). N_2_ was used as a carrier gas with a flow rate of 1.5 ml min^-1^. The injector and detector temperatures were set at 220°C. A molar ratio of 3:1 was used to convert C_2_H_2_ reduced to N_2_ fixed [61]. The annual biological N fixation rates for different aged stands were calculated by converting the measured N fixation rates (μmol m^-2^ d^-1^) per hectare and multiplying them by the average length of the growing season (120 days) of the study area. The annual N fixation rates for non-measured periods were linearly interpolated from sequential measured values.

### N_2_O fluxes

N_2_O fluxes between the soil and the atmosphere were measured in the study areas (Table 1) during the summer of 2013 [13] by using the static chamber method [62]. N_2_O flux measurements were done once a month in June, July and August on polyvinyl chloride collars (6 collars per plot; diameter 0.22 m and height 0.05 m). The gas samples taken from chambers (height = 0.24 m and diameter = 0.2 m) were analyzed by an Agilent Gas Chromatograph model 7890A (GC, Agilent Technologies, USA) equipped with an autosampler (Gilson GX-271 Liquid Handler) by using a Flame Ionization Detector. Samples were analyzed using six-point standard curve [62]. The N_2_O fluxes were calculated by using linear regression fitted to time and concentration change inside the chamber headspace. The annual N_2_O fluxes for different aged stands were calculated by converting the measured amount of N_2_O (mg m^-2^ s^-1^) per hectare and multiplying them by the average length of the growing season (120 days) of the study area. The annual N_2_O fluxes for non-measured periods were linearly interpolated from sequential measured values.

### N cycling genes

The number and intensity of microbial N cycling genes were detected from the study areas (Table 1) where the fire had occurred 2, 60 and 152 years ago [37]. There were 3 plots in each age class, and 5 soil samples per plot were collected at the interface of the humus and the mineral soil from 0.25 m by 0.25 m quadrats. The samples were mixed, transferred to 1.5-ml Eppendorf vials, frozen at -180°C in liquid nitrogen and transported to the laboratory on dry ice for subsequent DNA isolation. The extracted DNA was labeled and hybridized on the GeoChip 4.0 microarray, containing probes originating from fungi, bacteria, archaea and virus, as previously described [35]. Signal intensity of each spot was scored as positive and retained if the signal-to-noise ratio (SNR), calculated as (signal mean—background mean)/background standard deviation, was ≥2.0 [35]. The number and intensity of microbial genes associated with N cycling were analyzed. In total, 21 genes involved in N cycling were detected consisting of 2592 different kinds of probes.

### N leaching

A long-term average value of 0.5 kg ha^-1^ yr^-1^ was used for the N leaching for years 1980–2011 based on long-term studies on N leaching done in forested mineral soil-dominated catchments in Lapland [63, 64]. During these years, on average 14% of N deposition was lost by leaching. This ratio was used as the leaching estimate for the years 1850–1980. Nitrogen leaching was expected to increase after the forest fire. The magnitude and duration of increased N leaching after the forest fire was expected to be of the same order of magnitude as after clear-cutting [65], Table 4. Increases in total N export rates have been shown to be similar between harvested and burnt boreal catchments [10], and the impacts in leaching losses can be detected for the first ten years after treatment [65].

**Table 4:** Increase in nitrogen leaching (%) compared to natural background leaching values during the first ten years after forest fire [65]. The magnitude and duration of increased nitrogen leaching after forest fire was expected to be the same order of magnitude than after clear-cutting.

Years after fire	Increase in N leaching (%)
1	73
2	63
3	63
4	59
5	48
6	27
7	25
8	15
9	12
10	1

### Calculations and statistical analyses

Total N balance of the ecosystem was calculated as follows:

ΔS+ΔB=(DN+FN)−(EN+LN),

where Δ*S* is a change of N pools in the soil, *ΔB* is the change of N pool in biomass, *DN* is the atmospheric deposition of N, *FN* is the N-fixation, *EN* is the emission of N_2_O and *LN* is N losses by leaching. All units are kg ha^-1^ yr^-1^.

Monte Carlo simulations were performed to calculate the uncertainty associated with the estimates of N inputs and outputs. This method has been commonly used in quantifying uncertainty in nutrient balances [66]. The values of N fluxes (deposition, N fixation, leaching and N_2_O fluxes) were randomly selected from the normal distribution with given mean and standard deviation. We assumed that all the model parameters are mutually independent and follow normal distributions. The simulation was run 20 000 times, each time with a different input values. These results allowed computation of standard deviations and uncertainty estimates.

A linear mixed model was used to test for differences in biomass, N pools and N fluxes (biological N-fixation and N_2_O fluxes) across the fire chronosequence. Time since the last fire was used as a fixed factor and plot as a random factor. Variance was broken into inter-plot and intra-plot components in the mixed model thus avoiding possible errors introduced due to pseudoreplication in spatial sampling in ecological studies [67, 68, 69]. The biomass and N pools of vegetation were measured from the same plots at three year intervals. Because these two time periods are correlated, measurement time was used as a repeated factor in the analysis. The Bonferroni test was used in multiple comparisons between the age classes over the study period. Differences were considered statistically significant when P was < 0.05. A linear regression analysis was used to examine the relationship between biomass N content and the time elapsed since the fire. Statistical tests were performed using IBM SPSS version 23 (IBM Corp, Armonk, NY, USA).

## Results

### Changes in N pools

**Fig 1:**
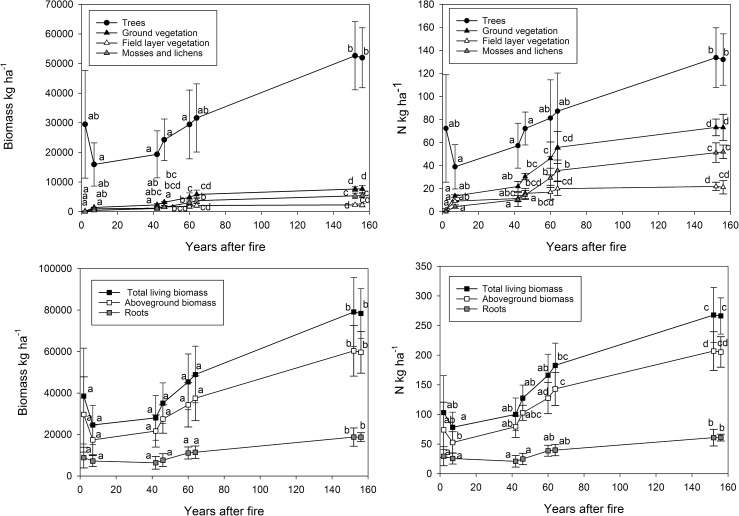
The average (±SE) biomass and nitrogen pools (kg ha^-1^) in above-ground tree biomass, field layer vegetation (vascular plants), bottom layer vegetation (mosses and lichens), ground vegetation (sum of bottom and field layer vegetation), total above-ground biomass, root biomass and total living biomass 2, 5, 42, 45, 60, 63, 152 and 155 years after the forest fire. Different letters indicate statistically significant differences (p < 0.05) between the age classes.

The biomass and its N pools increased with time since the fire (Fig 1, S1 Fig). The N pools in total living biomass increased by 2.5-fold (from 102 kg ha^-1^ to 260 kg ha^-1^) during the 155 post-fire years. This implies that N accumulated in biomass at an average annual rate of 1.02 kg ha^-1^. The stands where the fire had occurred ≤60 years ago had significantly lower (P < 0.05) biomass and N pools in total living vegetation compared to the oldest stands (152 and 155 years since the last fire occurrence).

The N pool in above-ground tree biomass was at its lowest 5 years after the fire (38 kg ha^-1^) after which it steadily increased, being 126 kg ha^-1^ when 155 years had elapsed from the fire (Fig 1). The bottom layer vegetation (mosses and lichens) was totally destroyed in the fire, and also the biomass (167 kg ha^-1^) and N pools (1.6 kg ha^-1^) of the field layer vegetation (vascular plants) were negligible in the recently burnt areas (Fig 1). In the 155-year-old stand, the biomass and N pool were 5450 kg ha^-1^ and 52 kg ha^-1^, respectively, in the bottom layer vegetation, and 2225 kg ha^-1^ and 21 kg ha^-1^, respectively, in the field layer vegetation. Also, the belowground biomass and N pools increased significantly with time after the fire. However, a relatively larger proportion of the biomass N pool was stored in the roots (30%) in the young age classes (2 and 5 years after fire) than in the older forests (20–23%).

**Fig 2:**
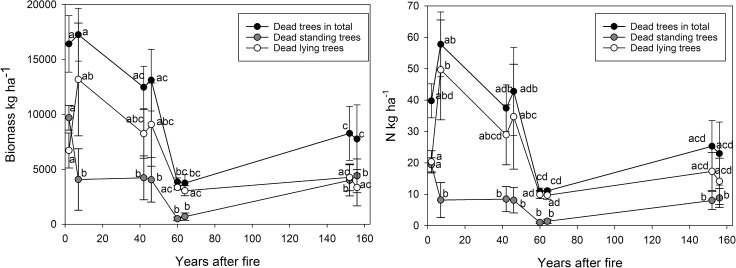
The average (±SE) biomass and nitrogen pools (kg ha^-1^) in dead trees 2, 5, 42, 45, 60, 63, 152 and 155 years after the forest fire. Different letters indicate statistically significant differences (p < 0.05) between the age classes.

The biomass and N pools of the dead trees were significantly higher shortly after the fire than in the older age classes (Fig 2). The biomass of standing dead trees decreased and the biomass of lying dead trees increased with time since the fire.

**Fig 3:**
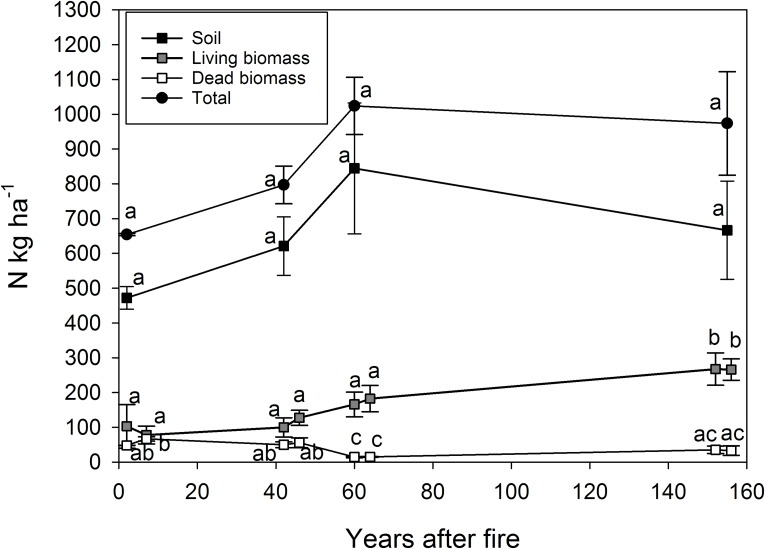
The average (±SE) nitrogen pools (kg ha^-1^) in different soil horizons 2, 42, 60 and 155 years after forest fire. Different letters indicate statistically significant differences (p < 0.05) between the age classes.

**Fig 4:**
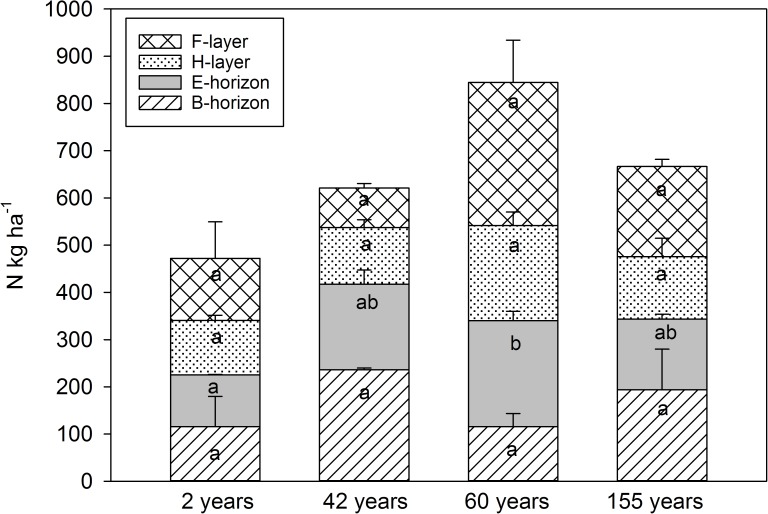
The average (±SE) nitrogen pools (kg ha^-1^) in soil (F-, H-, E- and B-horizons), living and dead biomass and total ecosystem along a 155-year fire chronosequence. Different letters indicate statistically significant differences (p < 0.05) between the age classes.

Total soil N pool was 29% smaller in areas where fire occurred 2 years ago (472 kg ha^-1^) compared to areas where fire occurred 155 years ago (667 kg ha^-1^). However, due to large within-plot variation, the total soil N pools did not differ statistically significantly between the age classes (Figs 3 and 4). When compared to changes between different soil layers, only the N pools in the E-horizon were significantly lower (P < 0.05) in the areas where the fire occurred 2 years ago compared to the areas where the fire occurred 60 years ago (Fig 3). Soil was the largest N pool in all forest age classes accounting for 72, 78, 82 and 68% of the total ecosystem N pools 2, 42, 60 and 155 years after the fire, respectively. The results suggest that N accumulated in the soil at an average rate of 1.26 kg ha^-1^ yr^-1^ during the 155 years period.

Two years after the fire, total ecosystem N pool was 654 kg ha^-1^ of which 20% was in the living vegetation, 8% in the dead biomass and 72% in the soil (Fig 4). 155 years after the fire, total N pool was 974 kg ha^-1^, with 28% in the living vegetation, 4% in the dead biomass and 68% in the soil. Ground vegetation accounted for only 0.3% of the ecosystem N pools 2 years after the fire but its share increased to 7% in the plots burnt 155 years ago.

### N cycling genes

In total, 2592 gene probes involved in N cycling were detected across the three areas (Table 5). The 2-year-old area after the fire had the highest number of detected probes (2109 ± 9; mean ± standard deviation) followed by the 152-year-old area (2039 ± 9; mean ± standard deviation) and the 60-year-old area (1895 ± 9; mean ± standard deviation). The total number of probes were significantly lower at the 60-year-old area than that at the 2-year-old area (*P* < 0.01), but there were no differences between the 2-year-old and 152-year-old areas. The genes (*narG*, *nirK*, *nirS* and *nosZ*) encoding enzymes for denitrification were more abundant (*P* < 0.05) at 2-year-old area than at 60-year-old area (Table 5). Also, the number and intensity of *nifH* gene related to N-fixation, and *NiR* and *narB* genes related to assimilatory N reduction were greater (*P* < 0.05) at 2- and 152-year-old areas than 60-year-old area.

**Table 5:** Number and intensity of genes involved in N processes. The values represent the mean and standard deviation of three replicates from each age class after fire. Different letters indicate statistically significant differences (p < 0.05) between the age classes. Genes showing significant differences among age classes are bolded.

Gene	Function	No. of genes			Gene intensity		
		2 years	60 years	152 years	2 years	60 years	152 years
*amoA*	Nitrification	5 ± 1 a	4 ± 1 a	3 ± 1 a	0.821 ± 0.10 a	0.554 ± 0.06 a	0.539 ± 0.18 a
*amoA_quasi*	Nitrification	22 ± 3 a	22 ± 3 a	19 ± 1 a	0.714 ± 0.08 a	0.727 ± 0.10 a	0.623 ± 0.01 a
*hao*	Nitrification	19 ± 1 a	16 ± 2 a	17 ± 2 a	0.830 ± 0.02 a	0.741 ± 0.10 a	0.752 ± 0.08 a
*cnorB*	Denitrification	8 ± 1 a	7 ± 1 a	8 ± 1 a	0.912 ± 0.12 a	0.849 ± 0.05 a	0.918 ± 0.10 a
***nirK***	Denitrification	163 ± 3 a	150 ± 5 b	151 ± 3 b	0.823 ± 0.01 a	0.766 ± 0.03 b	0.766 ± 0.02 b
***nirS***	Denitrification	155 ± 2 a	131 ± 13 b	150 ± 1 a	0.776 ± 0.01 a	0.656 ± 0.06 b	0.745 ± 0.01 a
*norB*	Denitrification	43 ± 2 a	36 ± 4 a	40 ± 2 a	0.806 ± 0.04 a	0.701 ± 0.07 a	0.765 ± 0.03 a
***nosZ***	Denitrification	251 ± 6 a	224 ± 7 b	246 ± 6 b	0.777 ± 0.02 a	0.691 ± 0.02 b	0.758 ± 0.02 b
***narG***	Denitrification	459 ± 6 a	428 ± 17 b	446 ± 3 ab	0.892 ± 0.01 a	0.817 ± 0.03 b	0.860 ± 0.01 ab
*gdh*	Ammonification	77 ± 2 a	73 ± 5 a	82 ± 3 a	0.773 ± 0.01 a	0.734 ± 0.06 a	0.806 ± 0.03 a
***ureC***	Ammonification	253 ± 5 a	234 ± 13 a	244 ± 2 a	0.856 ± 0.01 a	0.794 ± 0.04 b	0.824 ± 0.01 ab
*hzo*	Anammox	5 ± 1 a	4 ± 1 a	5 ± 2 a	0.602 ± 0.07 a	0.555 ± 0.12 a	0.551 ± 0.17 a
*hzsA*	Anammox	2 ± 1 a	2 ± 1 a	2 ± 1 a	1.008 ± 0.05 a	0.928 ± 0.07 a	0.787 ± 0.18 a
*napA*	Dissimilatory N reduction	79 ± 3 a	69 ± 5 a	79 ± 3 a	0.750 ± 0.03 a	0.664 ± 0.05 a	0.757 ± 0.02 a
*nrfA*	Dissimilatory N reduction	75 ± 3 a	74 ± 2 a	76 ± 2 a	0.760 ± 0.03 a	0.741 ± 0.02 a	0.770 ± 0.04 a
***narB***	Assimilatory N reduction	17 ± 1 a	14 ± 1 b	18 ± 1 a	0.918 ± 0.01 a	0.785 ± 0.05 b	0.924 ± 0.03 a
*nasA*	Assimilatory N reduction	61 ± 1 a	55 ± 5 a	59 ± 3 a	0.807 ± 0.02 a	0.726 ± 0.06 a	0.778 ± 0.03 a
***NiR***	Assimilatory N reduction	22 ± 1 a	17 ± 1 b	22 ± 1 a	0.738 ± 0.05 a	0.610 ± 0.02 b	0.756 ± 0.03 a
***nirA***	Assimilatory N reduction	20 ± 2 a	19 ± 1 a	18 ± 2 a	0.787 ± 0.05 a	0.790 ± 0.04 a	0.746 ± 0.07 ab
***nirB***	Assimilatory N reduction	47 ± 2 a	42 ± 1 a	45 ± 3 a	0.876 ± 0.03 a	0.787 ± 0.02 b	0.834 ± 0.03 ab
***nifH***	N fixation	328 ± 3 a	270 ± 20 b	309 ± 12 a	0.750 ± 0.01 a	0.625 ± 0.04 b	0.715 ± 0.03 a

### Nitrogen balance

**Fig 5:**
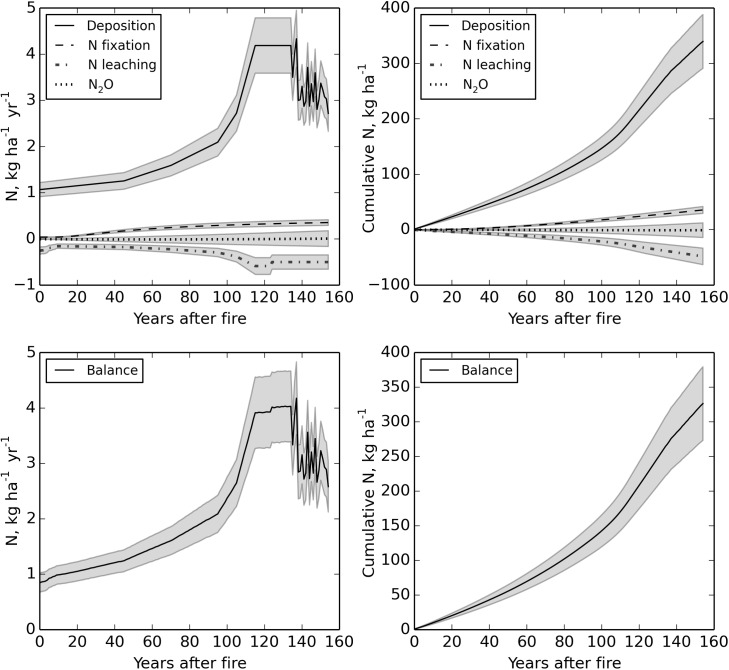
Annual and cumulative (kg ha^-1^) nitrogen input (atmospheric deposition and N-fixation), nitrogen output (leaching and N_2_O fluxes) and nitrogen balance along a 155-year fire chronosequence. Grey areas indicate the standard deviation which is based on 20 000 Monte Carlo iterations.

During 155 years, the atmospheric deposition of N was estimated to be 340 kg ha^-1^ (2.2 kg ha^-1^ yr^-1^) and it varied from 1.1 to 4.3 kg ha^-1^ yr^-1^ (Fig 5). The N inputs from N-fixation were 35.7 kg ha^-1^ (0.23 kg ha^-1^ yr^-1^) during 155 years. The N fixation rates increased with time after fire being 2.5, 6.6, 4.7 and 11.2 μmol m^-2^ d^-1^ respectively in the 5-, 45-, 63- and 155-year-old stands. On an annual basis this was equivalent to 0.08, 0.22, 0.16 and 0.38 kg ha^-1^ yr^-1^ in the 5-, 45-, 63- and 155-year-old stands, respectively. The N-fixation was significantly larger (P < 0.05) in the oldest age class compared to the other age classes. N-fixation constituted 9% of the total N input during the 155-year life span of the forests. The proportion of N-fixation of the total N input varied from < 1% to 15% during the 155 post-fire years.

During 155 years, the estimated N export by leaching was on average 0.31 kg ha^-1^ yr^-1^ (range 0.16–0.59 kg ha^-1^ yr^-1^) (Fig 5). The measured N_2_O fluxes were negligible (≤ 0.01 kg ha^-1^ yr^-1^) compared to the other N fluxes, and the oldest stand where the fire had occurred 155 years ago was a small sink of N_2_O (0.008 kg ha^-1^ yr^-1^). N_2_O fluxes, however, did not differ significantly between the forest successional stages. Total cumulative N output (49.0 kg ha^-1^) during the 155 post-fire years was small, accounting for only 13% of the input.

The forests were N sinks in all age classes, the annual N accumulation rate in soil and biomass being 2.28 kg ha^-1^ yr^-1^. These observed changes in ecosystem N pools were consistent with the computed average (mean ± SD) N balance 2.11 ± 0.34 kg ha^-1^ yr^-1^ over the 155-year post-fire period (Fig 5). The N balance varied from 0.84 to 4.18 kg ha^-1^ yr^-1^. Based on the Monte Carlo simulation, the estimated relative uncertainty (coefficient of variation) was 16% for N balance. The most important source of uncertainty for the N balance computation was associated with deposition (Fig 5).

## Discussion

This study is among the few [8, 14, 70] that have studied the long-term effects of fire on the pools, fluxes and balance of N in boreal forests, and to our knowledge, none of them have been conducted in Fennoscandian subarctic Scots pine forests. The results indicate that a large part of the living biomass (51%) and its N pools (61%) are destroyed in non-stand-replacing, intermediate-severity fires. The biomass and N accumulated more slowly in the bottom layer than in the field layer after the fire. The biomass and N pools of the bottom layer vegetation exceeded those of the field layer vegetation about 60 years after the fire. Previous studies have also indicated that mosses recover slowly from the fire [33, 71], and it takes more than 50 years until the coverage of feather mosses reaches the pre-fire levels [33, 71]. The biomass and N pools of ground vegetation in the oldest age classes were about two times higher than those reported for southern boreal Scots pine forests [72, 73]. Ground vegetation has typically greater significance in nutrient dynamics in northern than in southern Finland [73].

Dead trees constituted up to 7% of total ecosystem N pool during the initial successional stages after the fire. In general, post-fire forest ecosystems contain a large amount of standing and downed woody debris [74, 75, 76]. The biomass and N pools of lying dead trees increased with time since the fire probably because standing dead trees were weakened by decay and fell down. Dead wood biomass, as generally observed in boreal forests [74, 75] seemed to follow a U-shaped pattern during the succession of the forest stands. The early stage of forest development is characterized by relatively high amounts of woody debris generated as a result of disturbance. The amount of woody debris declines with time as the forest regenerates, and the woody debris input peaks again when mature stands senesce. The biomass and N pools in dead trees were lowest 60 years after the fire which is consistent with the decomposition estimates according to which it takes about 60 years for Scots pine stems to decompose [77] and lose most of their N [54].

Most of the N (80–90%) in boreal forests is stored in soil [8, 14, 30, 49, 73], and the same also applies to our post-fire stands. Soil N pools at our study areas were within the range reported for boreal Scots pine dominated barren heath or xeric heath forests [72, 73, 78]. Total soil N pools have been reported to decrease or remain unchanged after fire [8, 24, 25, 40, 79]. The conflicting results may derive from differences in climatic conditions, site types and the intensity of the fire [14, 70, 79, 80]. Light or moderate forest fires have been reported to reduce soil N pools by 15–30% in boreal forests [14, 22, 71, 81] and by 22% in temperate forests (a meta-analysis by Nave et al. [17]. In this study, soil N pool was 29% smaller 2 years after the fire than 155 years after the fire, though this difference was not statistically significant. It is difficult to detect changes in soil N over time due to large spatial variability [66, 78]. During the fire, the severity of combustion is variable ranging from a light burning to an extensive combustion of surface soils layers which may further increase the spatial variation in soil N pools [8]. One reason why N pools of the O-horizon did not significantly change after the fire may be due to increased litter N input to the soil. This is because tree crowns are not consumed by surface fire, but tree mortality can be high and occur during several years after the fire [41]. The fact that the N pools of E-horizon were lowest 2 years after the fire may be because in the mineral soil layers close to the soil surface, the N pools may increase with time after the fire because black carbon and recalcitrant N associated with it are formed by fires, and they are highly resistant to decomposition. This black C and N is transported from the forest floor to deeper soil horizons by percolation [17, 40]. Our results are consistent with the results of Wirth et al. [70], who found that in Siberian coniferous forests non-stand replacing fires redistribute soil N from the organic into the mineral layer but do not significantly decrease total soil N pools.

Typically, 1–2 kg N ha^-1^ yr^-1^ is accumulated in the soil of boreal forests [82, 83]. Thus, the estimated N accumulation rate in the soil (1.26 kg ha^-1^ yr^-1^) in this study was at the lower end of this range. In general, soil N pools have been found to recover to pre-fire levels within 20–100 years [17, 25, 84, 85]. Total ecosystem N pools were 33% (320 kg ha^-1^) smaller in the recently burned than in the mature stands. Boby et al. [79] reported a 50% (900 kg ha^-1^) decline in total ecosystem N pools after stand-replacing fires in boreal forests of Alaska. After less severe fires, as in our study, the total ecosystem N has been reported to decrease by 10–20% [14, 86, 87].

N gains due to post-fire N-fixation have not been previously quantified in subarctic boreal forests. Our results are in agreement with previous findings that N fixation increases with succession in boreal forests [33, 83, 88]. This was probably due to the increase in the moss biomass after the fire because in boreal forests majority of N-fixing bacteria live in mosses and the number of free-living N-fixing bacteria in the soil is low [89]. Furthermore, a lower amount of available N and more favorable moisture conditions in the forest stands at late successional stage enhance the N-fixation [88, 90]. The measured N fixation rates in our study were lower than those reported in other studies for early (4–80 years) successional (0.1–1.0 kg ha^-1^ yr^-1^) and late (100–200 years) successional (0.4–2.1 kg ha^-1^ yr^-1^) boreal forests [29, 33, 91, 92]. This discrepancy may be due to lower coverage of feather mosses, shorter growing season and lower temperature regimes in our study areas [90, 93]. Furthermore, the above-mentioned studies [29, 33, 92] concern only the N fixation of the dominant bottom layer species (*Pleurozium schreberi*). Even if N deposition was quite low in our study area, it might have decreased the N fixation because it has been shown that the N fixation in mosses is reduced already by N deposition as low as 3 kg N ha^−1^ yr^−1^ [94]. The N fixation in boreal forest soils is usually considered to be negligible. This study indicates, however, that N fixation potentially exists also in the bare soil after the forest fire, and this was also manifested in the increased amount and intensity of N-fixing genes in the soil of the youngest age class. Potentially occurring N fixation in dead wood was not included in this study, though it may also slightly increase N input to forest stands [28]. On average, 9% of the N that was added to the ecosystem during the 155 post-fire years was from N-fixation. N fixation has been shown to play a more important role in the N budget of temperate [19, 95] and some North American boreal forests [96] where N fixation can contribute up to 70% of the total annual N input.

The calculated N balance (+2.11 kg ha^-1^ yr^-1^) was consistent with the observed changes in ecosystem N pools (+2.28 kg ha^-1^ yr^-1^). Our N balance results are comparable to those obtained in modeling studies in northern Sweden in which N accumulation rates were estimated to be 2.6 kg ha^-1^ yr^-1^ [97]. Berg & Dise [82] estimated that N accumulation in northern European forests ranges between 2.9 and 3.3 kg ha^-1^ yr^-1^ corresponding to 85% of the N input. Atmospheric deposition represented the major input of N in these forests. Deposition records suggest that N deposition has increased by 2–3 kg ha^-1^ yr^-1^ in our study areas during the past 155 years (Fig 5). Similarly, Hyvönen *et al*. [6] generated historical N deposition data based on the studies by Lövblad *et al*. [98] and Schöpp *et al*. [99], and reported that N deposition has increased from 1 to 4 kg ha^-1^ yr^-1^ in northern Sweden between the years 1900 and 2000. The elevated N deposition could have increased forest productivity and C sequestration because it has been estimated that ecosystem C stocks increase by 30–70 kg C ha^-1^ yr^-1^ per 1 kg N deposited ha^-1^ yr^-1^ in N-limited forests [6, 7]. In the future, increasing N deposition may alleviate N losses caused by more frequently occurring fires and thus contribute to forest’s capacity to sequester C, even though increasing N deposition may slightly reduce N fixation. Our estimate that 13% of N input was lost by leaching was the same order of magnitude than presented in earlier studies (<10–15%) for N-limited boreal forests [27, 82]. In N-limited boreal forests, N cycling is closed and leaching losses are small [27, 30] even after disturbances [100].

The abundance of genes associated with nitrification did not change with age after fire (Table 5), although nitrification has generally been observed to increase shortly after the fire because of increased pH, elevated NH_4_-N concentrations and charcoal-induced reductions in phenolic compounds [29, 38]. The number and intensity of genes related to denitrification and assimilatory N reduction, in turn, were significantly higher in the recently burned area than in the mature forest. Different N cycling genes have different sensitivities to environmental changes, and denitrifying genes (*nirS* and *nosZ*), have been shown to be the most sensitive to many environmental factors such as the availability of N [101]. Also, Taş *et al*. [102] found that genes responsible for denitrification and assimilatory N reduction were more abundant in burned than in unburned boreal soils in Alaska. The higher abundance of genes involved in denitrification in recently burned areas, however, did not lead to increased N_2_O fluxes [13]. This may be due to that both the N_2_O producing (*nirS* and *nirK*) and removing (*nosZ*) genes increased in recently burned plots. The oldest stand was a small sink for N_2_O which could be seen as low number and intensity of genes related to denitrification. In our study, genes responsible for N fixation were more abundant in recently burned and the oldest areas compared to 60-year old stand. The higher abundance soon after fire may be due to increases in soil pH and temperature after the fire [39] because the abundance of N-fixing bacteria and N fixation activity are positively related to pH and temperature [31, 34]. In contrast to our study, in a study performed in Alaska the abundances of genes involved in N fixation were not affected by the fire [102]. However, in our case, the abundance of genes involved in N fixation did not correlate significantly with N_2_ fixation measurements. The reason for this discrepancy may be that in acetylene reduction assay used in this study, the mosses were also included whereas gene microarray was performed using DNA extracted from soil. Mosses are known to be important in forest N fixation [28, 33]. However, the high N fixation gene abundance in soil indicates that N fixation in soil may also have role in the soil N cycling. The abundances of functional genes can serve as tools to predict the capacity of an ecosystem to carry out a given process or predict potential process rates [36, 103]. However, gene numbers do not necessarily provide information on real-time process rates because fluctuations in environmental conditions can cause rapid changes in process rates, but does not necessarily affect gene abundance [36, 103]. With recent technological advances, RNA-based methods will provide in the future better real-time picture on microbe-driven soil processes with reasonable costs.

Chronosequence method assumes that every study area has similar biotic and abiotic history and the only difference among areas is the time since fire [69, 104]. In practice, inherent differences among areas and other factors such as pre-fire conditions, fire behavior, or climatic conditions following the fire may cause variation, inaccuracy and even misinterpretation of results in chronosequence studies [69, 104]. Taking into consideration that our study areas had similar elevation, topography, microclimate, soil properties and vegetation, it is probable that much of the observed differences were due to the time elapsed since the last fire. The second factor causing uncertainty is that this study contained only one area per each age class and we did not had pre-fire data from the study areas. However, our measurement areas were relatively large in size (the total study area was 18 km^2^) and the measurement plots within each area were located at least 200–500 m from each other and thus cover the spatial variability in the area well. The limited number of replicates is a common drawback in this kind of comparative studies [69] which restricts making broad generalizations of the findings. In addition, forest nutrient budget studies contain uncertainties which are caused by spatial and temporal variation and inaccuracies related to used nutrient flux and biomass estimations [66, 67, 68]. The uncertainty caused by biomass estimations were probably small because the input variables (diameter and height) were measured from each tree on the study plots, and the used biomass models are developed especially to Finnish conditions and they are based on extensive empirical data [48]. Flux calculations assume constant concentrations between measurements, and interpolating linearly between measurements may cause inaccuracy in the budget calculations [66]. According to Monte Carlo simulations, the most important source of uncertainty for the N balance computation was associated with deposition which reflects annual and regional variation in precipitation.

## Conclusions

The results suggest that non-stand-replacing fires in sub-arctic pine forests decrease considerably the N pools in living vegetation and increase the N pools stored in dead biomass. However, it seems that changes in total ecosystem N pool are minor, because large N pool in the soil does not noticeably change. The N pools of living vegetation increase with time since fire and contribute an increasing proportion of total ecosystem N in older stands. The results show that in the studied subarctic ecosystems, atmospheric deposition represents the major input of N, and the N outputs are small, assuming that our assumptions about long-term leaching are correct. N-fixation depends on the successional stage after a forest fire. Based on the number and intensity of genes related to N-fixation as well as the measurements of N-fixation, it seems that N-fixation rates can be substantial on the surface of the humus layer after the fire. However, because most of the N fixation takes place in mosses, and moss biomass increases with post-fire succession, total ecosystem N fixation rates increase with time since fire. N-fixation is five times higher in mature forests than in recently burned areas. N_2_O fluxes are negligible (≤ 0.01 kg ha^-1^ yr^-1^) compared to the other N fluxes and do not significantly differ among post-fire age classes.

## Supporting information

S1 Fig Relationship between total living biomass (above- and belowground biomass), above-ground tree biomass and ground vegetation biomass (sum of bottom and field layer vegetation) and time since the fire. Relationship between the nitrogen pools of total living biomass, above-ground tree biomass and ground vegetation and time since the fire.
(TIF)

S1 File Data (the pools, fluxes and balance of nitrogen and nitrogen cycling genes). (XLSX)
