# Supplementary figures and images for: Nitrogen balance along a northern boreal forest fire chronosequence
Source: PLoS One. 2017 Mar 30;12(3):e0174720. doi: 10.1371/journal.pone.0174720 (PMC5373610; doi:10.1371/journal.pone.0174720)

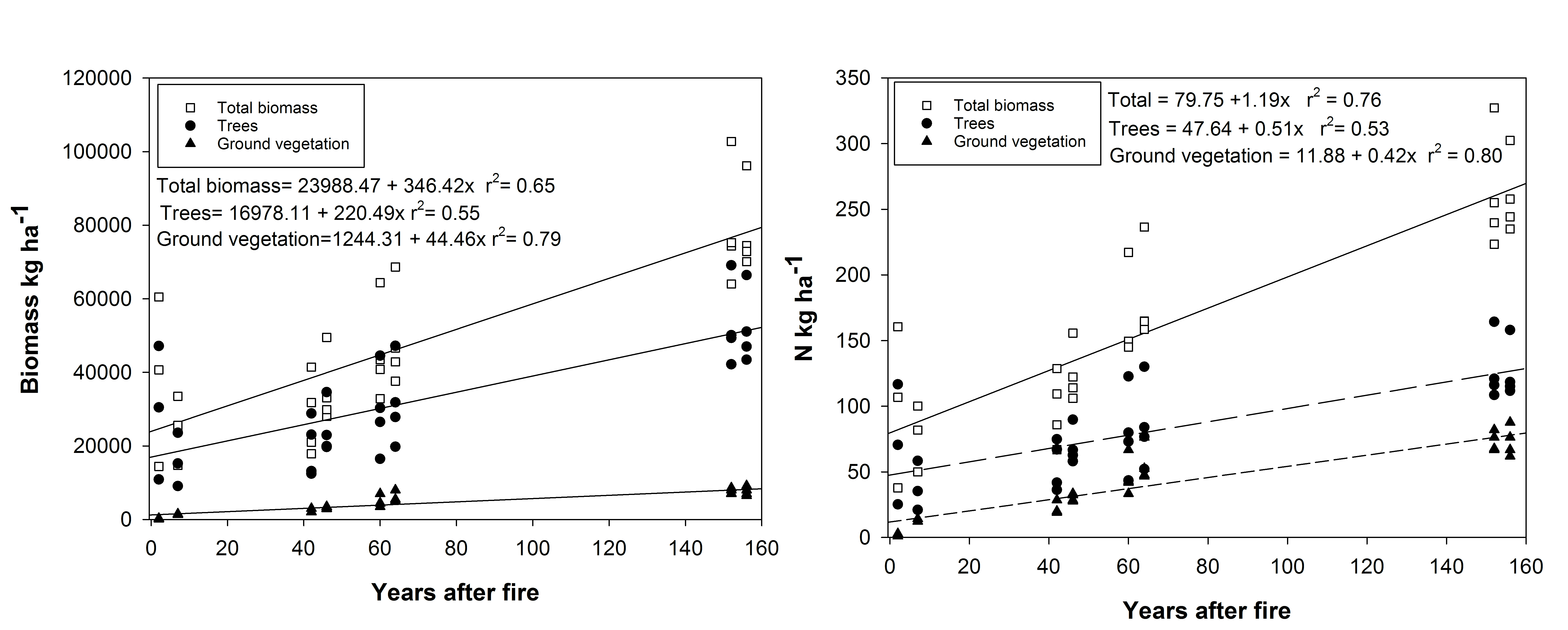

Supplement: S1 Fig — Relationship between the nitrogen pools of total living biomass, above-ground tree biomass and ground vegetation and time since the fire. (TIF) [file pone.0174720.s001.TIF]
